# The Experience of Empathy in Everyday Life

**DOI:** 10.1177/0956797621995202

**Published:** 2021-07-09

**Authors:** Gregory John Depow, Zoë Francis, Michael Inzlicht

**Affiliations:** 1 Department of Psychology, University of Toronto; 2 Department of Psychology, University of the Fraser Valley; 3 Rotman School of Management, University of Toronto

**Keywords:** emotions, interpersonal relationships, motivation, personality, sex differences, social cognition, theory of mind, well-being, open data, open materials, preregistered

## Abstract

We used experience sampling to examine perceptions of empathy in the everyday lives of a group of 246 U.S. adults who were quota sampled to represent the population on key demographics. Participants reported an average of about nine opportunities to empathize per day; these experiences were positively associated with prosocial behavior, a relationship not found with trait measures. Although much of the literature focuses on the distress of strangers, in everyday life, people mostly empathize with very close others, and they empathize with positive emotions 3 times as frequently as with negative emotions. Although trait empathy was negatively associated only with well-being, empathy in daily life was generally associated with increased well-being. Theoretically distinct components of empathy—emotion sharing, perspective taking, and compassion—typically co-occur in everyday empathy experiences. Finally, empathy in everyday life was higher for women and the religious but not significantly lower for conservatives and the wealthy.

Empathy—understanding, sharing, and caring about the emotions of other people—is important for individuals, fundamental to relationships (Kimmes et al., 2014), and critical for large-group living (Decety et al., 2016). Unfortunately, evidence suggests that empathy is on the decline (Konrath et al., 2011). Despite the wealth of experiments on empathy, we lack a descriptive account of how it is experienced in daily life. Experimentation is critical, but description is also necessary and has too often been neglected in psychology (Rozin, 2001). Much of what we know about empathy has been established in laboratory settings—meaning that we do not yet know whether these findings are generalizable to the real world.

The current study advances our understanding of empathy by moving it “into the wild”—asking people directly about their experiences of empathy in everyday life. We aimed to answer important, unaddressed questions. How often do people perceive empathy opportunities, and how often do they empathize? What types of empathy do people most often report feeling? Whom do people most often empathize with? In addition, we tested preregistered hypotheses that empathy is associated with increased subjective well-being and increased rates of prosocial behavior in daily life. We further examined relationships between empathy in daily life and individual-differences measures, including gender, religiosity, income, and political orientation.

## Describing Empathy in Daily Life

The inconsistency with which empathy has been defined has been rightfully criticized (Hall & Schwartz, 2019), but many researchers think empathy involves sharing someone’s emotion (an emotional process), taking someone’s perspective (a cognitive process), and feeling compassionate and wanting to help (a motivational process). There is no consensus about whether these components—emotion sharing, perspective taking, and compassion—are distinct or different sides of the same empathy construct (Zaki, 2014). There is evidence that they can be differentiated (Winter et al., 2017). However, these components may be tightly intertwined for most people, even if they are technically dissociable (Fiske, 2009; Zaki & Ochsner, 2012). The lack of clarity around whether the components of empathy co-occur contributes to the general confusion about how to define empathy (Batson, 2009; Cuff et al., 2014). Unfortunately, few studies have actually examined empathy in daily life to test the perceived co-occurrence of emotion sharing, perspective taking, and compassion.

Here, we addressed limitations of prior research by improving ecological validity (Schreiter et al., 2013) while examining multiple emotions across a range of social contexts (Morelli et al., 2014). Contexts studied in the lab may or may not match the contexts in which empathy occurs in daily life. For example, many studies on empathy have examined how people empathize with strangers, although evidence suggests that people are biased to feel more empathy for close others (Cikara et al., 2011; Meyer et al., 2013). With the current approach, we were able to examine the closeness of real-life empathy targets.

In addition, research on empathy has typically focused on observed pain. If unchecked, a nonrepresentative focus in the research can distort our understanding of empathy. For example, a meta-analysis of the neuroscience of empathy suggested that the dorsal anterior cingulate cortex and bilateral anterior insula formed part of a core empathy network (Fan et al., 2011), but more recent work has shown that this consistent activation may be due to the overrepresentation of studies showing empathy for pain in the functional-MRI literature (Morelli et al., 2014). Without an account of how empathy is generally experienced in daily life, we cannot be certain that other conclusions drawn from the literature do not face similar issues. Increasingly, empathy for positive emotions is recognized as important (Andreychik & Migliaccio, 2015; Morelli et al., 2015; Telle & Pfister, 2012). The current study focused on whether people report experiencing empathy more often in response to positive or negative emotions and whether these lead to differential outcomes.

## Correlates of Empathy in Daily Life

Feeling empathy may affect one’s own subjective well-being, although evidence is mixed. Some researchers have argued that empathy leads to increased well-being, whereas others posit that it leads to burnout and social withdrawal (for a review, see Konrath & Grynberg, 2016). Some authors have contended that empathy is neither positive nor negative but results in either compassion or personal distress, which have distinct phenomenology and consequences (Singer & Klimecki, 2014). It is not clear whether the complexity of real-life social interactions will respect the boundaries of these taxonomies. In this study, we examined real-life perceptions of empathy to determine how different empathy experiences affect subjective well-being in the daily lives of the general population.

**Statement of Relevance**

Empathy allows us to connect with other people by taking their perspective, sharing their emotions, and feeling compassion for them. This ability, coupled with unprecedented access to the emotional experiences of other people, should lead to increased social connectedness. Instead, self-reports of empathy are declining, and loneliness is on the rise. Here, we provide an important step to combating these trends by understanding how individuals perceive and respond to the deluge of empathy cues to which they are exposed in everyday life. We found that people often report opportunities to provide as well as receive empathy. In the current study, we used a novel description and rich, representative data set of empathy experiences in everyday life across a range of social and emotional contexts. We tested connections between empathy and subjective well-being as well as empathy and real-life prosocial acts while bringing data to bear on a number of important and unresolved questions.

In the lab, feeling empathy often promotes prosocial behavior (for a review, see Davis et al., 2015), but there is also evidence that empathy can promote antisocial behaviors such as hyper-competition (Pierce et al., 2013) and even immorality (Batson et al., 1995). In the current study, we thus examined whether—and under what circumstances—empathy is associated with prosocial behavior in the context of everyday life.

An experience-sampling approach can uncover relationships that trait measures miss because it reduces recall bias and allows us to parse within-subjects variability from between-subjects variability (Runyan et al., 2019; Shiffman et al., 2008). The current study allowed us to compare the predictive power of trait and state measures of empathy in predicting prosocial behavior and subjective well-being.

When individuals engage in moral acts, they may feel licensed to engage in subsequent immoral acts, an effect known as moral licensing (Merritt et al., 2010). Although lab-based research on this effect has been underpowered (Blanken et al., 2015) and subject to publication bias (Kuper & Bott, 2019), experience-sampling research has found that performing a moral act at Time 1 has a “licensing” effect, which makes a moral act at Time 2 less likely to occur (Hofmann et al., 2014). Here, we tested whether empathy shows a similar licensing effect or whether, instead, empathy at Time 1 might increase the chance of experiencing empathy again at Time 2—as a sort of “empathy-facilitation” effect.

## Who Feels Empathy in Daily Life

Empathy varies across demographic variables including geographic region (Bach et al., 2017), age (O’Brien et al., 2013), ethnicity (Chiao & Mathur, 2010), education (Ward et al., 2012), income (Stellar et al., 2012), and gender (Christov-Moore et al., 2014). Therefore, we used quota sampling to obtain a sample that was representative on these parameters. Given prior findings, we performed preregistered tests of the hypothesis that women are more empathic than men. Research also suggests that religiosity (Watson et al., 1984) and liberalism (Hasson et al., 2018) are associated with increased empathy, whereas income is associated with reduced compassion (Stellar et al., 2012). We thus performed nonpreregistered analyses of these relationships.

## Method

### Study design

Researchers have called for increased ecological validity (Schreiter et al., 2013) and demonstrated that experience sampling may uncover associations that global reports miss (Billeke et al., 2015; Runyan et al., 2019). Thus, in the current study, we took a mixed-methods approach, combining established trait measures of empathy with experience sampling. We add to a small list of studies that have examined empathy with experience sampling or ecological momentary assessment (Grühn et al., 2008; Nezlek et al., 2001). This allowed us to explore within-subjects differences that have historically been neglected in empathy research (Duan & Hill, 1996) and to examine causal heterogeneity, which is an overlooked problem in psychology more generally (Bolger et al., 2018).

### Procedure

#### Sampling strategy

**Fig. 1.**
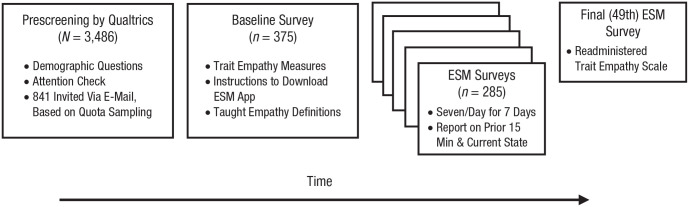
Flowchart of procedure. For the experience-sampling method (ESM) surveys, 285 participants completed at least one experience-sampling survey, but 39 participants were excluded for having fewer than seven surveys according to our preregistration, leaving a final sample size of 246 to be analyzed.

Given that empathy varies across demographic variables (Bach et al., 2017; Chiao & Mathur, 2010; Christov-Moore et al., 2014; O’Brien et al., 2013; Stellar et al., 2012), we used quota sampling to obtain a sample representative of the U.S. population on key demographic parameters (Fig. 1). To this end, 3,486 potential participants answered a demographic questionnaire, on which they also provided informed consent about participating in a “daily interactions” study. In cooperation with the survey company Qualtrics (www.qualtrics.com), we selected 841 individuals from this pool of respondents to invite, using quota sampling to match our sample to U.S. census data on sex, ethnicity, education, geographic region, income, and age. In the current study, we use the term *representative* to mean representative on these six parameters.

Although ours was not a true random sample, this approach represents a significant improvement over convenience samples used in much of psychological research. A total of 376 participants responded to our e-mail to complete the baseline survey, and 285 went on to download an app and complete experience sampling (Fig. 1). Given logistic demands of the study, we planned for high attrition in our sampling strategy.

We ran a number of simulations (see Fig. S1 at https://osf.io/zd6wv/) to determine the sample size required to accurately estimate the frequency of empathy in daily life. We also consulted previous research on the sample size required to enter the “corridor of stability” for parameter (Maxwell et al., 2008) and correlation (Schönbrodt & Perugini, 2013) estimates. We preregistered our intention to recruit a sample of 300 participants, but simulations and prior research suggested that we would enter the corridor of stability between 200 and 300 participants. Practical considerations, mainly financial, halted collection at 285 participants.

Sensitivity analyses further suggested that this sample size has 80% power to detect between-subjects effects (*d*) of 0.33 or greater. At the survey level, we had 80% power to detect between- and within-subjects effects of 0.18 and 0.08, respectively.

Following our preregistration, we removed participants who answered fewer than seven experience-sampling surveys, resulting in a final sample (*N* = 246) that was representative of the population (see Figs. S2–S7 at https://osf.io/zd6wv/) as well as adequately powered to make precise estimates about the frequency of empathy and its correlation with prosocial behavior and well-being. During experience sampling, participants answered a total of 7,343 surveys, providing a rich and ecologically valid data set. We preregistered our hypotheses and design (https://osf.io/aeqgn) and provide open access to materials, code, and data for transparency and the benefit of other researchers (https://osf.io/y3ud7).

#### Data collection and compensation

After participants were recruited, they completed a baseline survey that included demographic information, such as socioeconomic status, religiosity, and political orientation, as well as the Interpersonal Reactivity Index (Davis, 1983), the Empathy Index (Jordan et al., 2016), the empathy-selection task (Cameron et al., 2019), a Big Five measure of personality (Soto & John, 2017), and other measures (see Material S1 at https://osf.io/zd6wv/).

Because the definition of empathy is not always clear among researchers (Batson, 2009; Hall & Schwartz, 2019) or laypeople (Hall et al., 2021), our baseline survey provided a glossary of terms that participants would later report on. We defined what we meant by empathy opportunity, empathy, emotion sharing, perspective taking, compassion, and prosocial acts (see Material S2 at https://osf.io/zd6wv/). Providing definitions ensured that the participants would be answering questions about the same concepts, although there would be a possible trade-off of introducing demand. The baseline survey also included instructions to download the experience-sampling app MetricWire (2019, Version 4.1.2).

We used the MetricWire app to prompt participants seven times a day between 10 a.m. and 10 p.m. for 1 week to complete short, signal-contingent surveys (see Material S3 at https://osf.io/zd6wv/). Survey triggers occurred semirandomly (i.e., randomly within 90-min windows with minimum 15-min gaps) and expired after 20 min. We asked participants about their current happiness and sense of purpose as a measure of subjective well-being. We also asked whether, in the past 15 min, they (a) had performed a prosocial act, (b) had an opportunity to empathize, and (c) had an opportunity to receive empathy.

If they indicated that they had had an opportunity to give or receive empathy, we probed for further details. For example, we asked how close they were to the other person involved. When participants had an opportunity to empathize themselves, we asked them to rate the valence of the emotion they observed on scale ranging from 1 (*extremely negative*) to 7 (*extremely positive*). We asked whether they had actually experienced empathy at the time and, if so, whether this experience involved emotion sharing, perspective taking, and compassion in turn. For each component that was reported, we asked about extent, difficulty, and confidence. Surveys involved branches that were effort matched so that participants would see the same number of questions regardless of their responses. This prevented participants from simply saying “no” to any question as a means to complete a survey more quickly.

Instead of the typical experience-sampling questions, the final experience-sampling survey readministered select trait empathy measures that had been previously completed at baseline to assess stability of trait empathy (see Material S3).

Participants earned a minimum of $18 (U.S.) for participating, along with a bonus of $7 if they completed 80% or more of the experience-sampling surveys. Answering 100% of the surveys would take about 2 hr total.

### Statistical analysis

We hypothesized that empathy would be related to subjective well-being and prosocial behavior in daily life, but there are many aspects of empathy. Thus, we preregistered an exploratory approach in which we tested each component as a single predictor and adjusted the *p* values of a given research question (e.g., daily empathy is associated with increased subjective well-being as a within-subjects effect) to control the false-discovery rate (FDR; Benjamini & Hochberg, 2007).

We used generalized mixed-effects models (multilevel logistic regression) for binary outcome variables such as empathy opportunity, prosocial behavior, and empathy. When the outcome variable was continuous, such as extent of empathy or subjective well-being, we used mixed-effects models (multilevel linear models). Observations were nested within participant, which were nested within survey day, so all multilevel models included a random intercept for both participant and survey day. Linear models were employed for tests of between-subjects effects predicting outcomes measured only once, such as testing whether demographic variables predicted the number of surveys answered.

For continuous outcome variables, we used participant-centered variables as predictors to test within-subjects effects and grand-mean-centered variables as predictors for between-subjects effects. For binomial predictors, we used dummy-coded (1 for yes, 0 for no) responses for testing within-subjects effects and grand-mean-centered average proportion of “yes” responses of a participant for testing between-subjects effects. As an illustration, a participant who reported empathy opportunities on 40% of surveys would receive a score of 0.40, which would be grand-mean centered and entered into the multilevel model as a single predictor nested within participant and survey day to test for between-subjects effects of the tendency to report empathy opportunities.

Data were analyzed in the R programming environment (Version 3.6.2; R Core Team, 2019). Multilevel models were constructed with the lmer and glmer functions using the *lme4* package (Version 1.1-14; Bates et al., 2015) and linear models with the lm function in the built-in R *stats* package. Model statistics were calculated with the summaryh function from the *hausekeep* package (Version 0.0.0.9003; Lin, 2019), including a validated effect-size *r* for fixed effects in multilevel models derived from *R*^2^ (Edwards et al., 2008) that has previously been used in the literature (e.g., Francis et al., 2020). For continuous data, β estimates indicate the model-estimated change in the dependent variable associated with a 1-unit change in the predictor. For binomial data, β estimates indicate the log odds ratio of a “yes” response holding all other covariates constant. Predictors were grand-mean or participant-mean centered as appropriate (Kleiman, 2018), and *p* values were corrected with the p.adjust function from the *stats* package following the FDR procedure (Benjamini & Hochberg, 2007). Given the richness of this data set, we do not present results of all preregistered analyses in the current article.^1^ Full survey materials are available on this study’s OSF page, along with deidentified data, analysis code, and supplemental material (https://osf.io/y3ud7).

## Results

### Empathy in everyday life

**Fig. 2.**
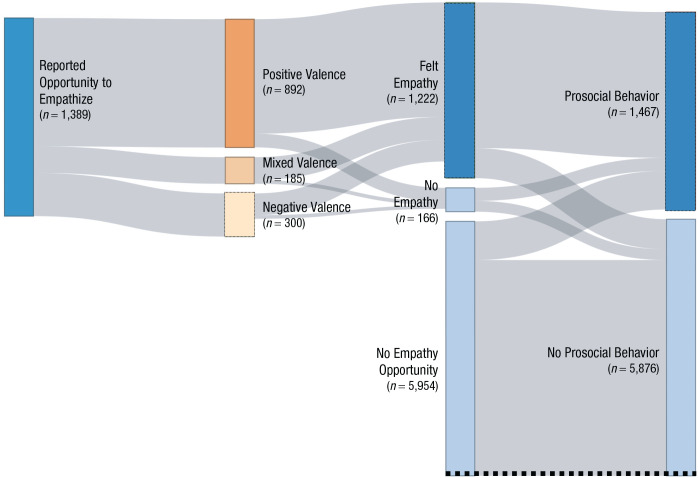
Sankey network diagram showing the number of each type of event. Beginning on the left, the diagram illustrates whether an empathy opportunity was reported. The valence ratings of reported opportunities and whether participants actually felt empathy are displayed next. Finally, the right side of the diagram displays whether a prosocial behavior was reported in the same 15-min time frame. Pathways are weighted to the number of surveys going through each pathway (except for the no-empathy/no-prosocial-behavior pathway, which has its width truncated as indicated by the dotted line).

Our data suggest that people commonly experience empathy in daily life. Examining the raw data, we found that individuals reported perceiving an empathy opportunity in the past 15 min across 19% of all surveys (see Fig. 2; *SD* = 39%, 95% confidence interval [CI] = [18%, 20%]) and reported perceiving an opportunity to receive empathy in the past 15 min across 12% of surveys (*SD* = 32%, 95% CI = [11%, 13%]). Participants had been informed that an opportunity to empathize was defined as any exposure to the emotions of other people (see Material S2), such as reading a sad status from a friend on social media or watching a stranger laugh. Looking at the mean of participant means yields similar rates (*M* = 21%, *SD* = 20%, 95% CI = [18%, 23%]; *M* = 13%, *SD* = 18%, 95% CI = [11%, 15%]), showing that these estimates are not unduly influenced by response-rate bias.

Using participant means from our data, we estimated that—over the course of a 12-hr day—an average individual may perceive 8.92 opportunities to empathize (*SD* = 9.93, 95% CI = [7.68, 10.16]) and perceive 5.71 opportunities to receive empathy (*SD* = 8.87, 95% CI = [4.60, 6.82]). These estimates may underestimate how often empathy opportunities are truly perceived, given that (a) participants were able to report on only a single empathy opportunity from the prior 15 min, whereas multiple opportunities may have occurred during that time period, and (b) participants may have forgotten or failed to report an opportunity to empathize. Alternatively, these estimates may overestimate the true perceived frequency because participants may feel demand to report empathy opportunities and empathy within the context of the study because of social desirability, or they may prefer to answer surveys when they have an opportunity to report. However, these initial estimates suggest that experiencing and receiving empathy occur frequently enough to have a significant psychological impact.

#### Missed opportunities

If participants reported no opportunity to empathize, they were later asked whether they had observed the emotions of another person in the same time frame. They responded affirmatively to this question on roughly 9% of occasions (*SD* = 29%, 95% CI = [9%, 10%]). On the basis of the definition that participants were taught and tested on at baseline (see Material S3), these emotional exposures were, by definition, opportunities to empathize. Therefore, participants missed numerous empathy opportunities in daily life, even when the cue was salient enough to be noticed. These findings are in line with research showing that empathy opportunities are often missed, for example, in physician–patient interactions (e.g., Giroldi et al., 2020; Morse et al., 2008).

When an opportunity was reported, participants reported actually feeling empathy 88% of the time (*SD* = 32%, 95% CI = [86%, 90%]). The same was true when participants reported an opportunity to receive empathy—they reported actually receiving empathy in 88% of these opportunities (*SD* = 23%, 95% CI = [87%, 90%]). These data suggest that people often experience empathy after noticing an opportunity but also miss many opportunities in daily life. These missed opportunities may help to reconcile the discrepancy between the very high rates of choosing to empathize that we observed in daily life and the tendency to avoid empathy that has been previously observed in the lab using the empathy-selection task (Cameron et al., 2019).

#### Components of empathy

**Fig. 3.**
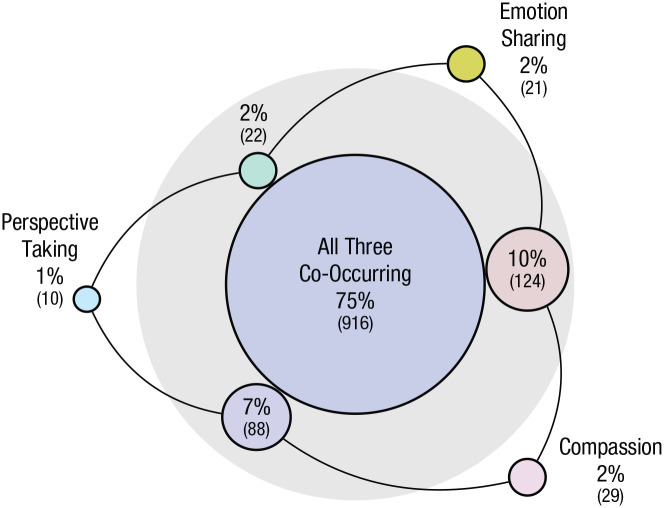
Components of empathy in daily life. The area of each circle reflects the relative percentage of the three components of empathy occurring individually or concurrently when empathy is reported. The large middle circle shows the 75% of occurrences in which all three facets co-occurred, and the three circles in the light gray ring reflect cases in which only two facets co-occurred. Not shown, on 1% of reports, participants said “no” to all three of the empathy components. Values in parentheses indicate numbers of empathy reports.

When engaging in empathy, which facets of empathy do people report? In the current study, the co-occurrence of the three different components was very high. All three components—emotion sharing, perspective taking, and compassion—were reported to occur together 75% of the time, and a single component occurred in isolation in only 5% of cases (see Fig. 3). Participants nearly always reported feeling compassion (94%), whereas perspective taking was the least frequently reported (85%). When participants reported a component, they were asked to rate the extent to which it occurred—for example, the extent to which they felt compassion or shared the person’s emotion. Component-extent ratings showed significant medium-size correlations (*r*) ranging from .45 to .49. These data provide evidence that theoretically distinct components of empathy tend to co-occur and interact during the complex social interactions of daily life (Zaki & Ochsner, 2012).

#### Contexts of empathy

Participants rated how close they were to the other person in each empathy experience on a scale ranging from 1 (*total stranger*) to 7 (*very close relationship*). In most cases (57%), participants reported on an opportunity to empathize with someone with whom they had a very close relationship. In only 6% of cases did participants report on an opportunity to empathize with a total stranger. Participants were not significantly more likely to empathize, *b* = 0.12, *SE* = 0.07, *z* = 1.74, *p* = .082, *r* = .03, as closeness increased. When empathy occurred, participants exhibited a small effect of empathizing to a greater extent as closeness increased, *b* = 0.09, *SE* = 0.02, *t*(1023) = 5.11, *p* < .001, *r* = .16. These results stand in contrast to the typical procedure used in empathy studies, in which the modal experience of empathy involves a stranger as the target.

In a similar vein—although “distress is the typical emotion of the object when the term empathy is used” (Preston, 2007, p. 436) in the literature—in our data, participants reported perceiving an opportunity to empathize with a positive emotion (65.8%) about 3 times as often as they reported on an opportunity to empathize with negative emotions (21.3%), and the remainder (12.9%) were mixed valence. Not only were positive emotions reported as empathy opportunities more often, but also participants reported empathizing to a greater extent as valence became more positive, *b* = 0.11, *SE* = 0.02, *t*(991) = 6.61, *p* < .001, *r* = .21, although the effect was small. Taken together, these data suggest that in daily life, empathy for positive emotions is a more common and more extensive experience than empathy for negative emotions, underscoring recent calls in the literature for more attention to this side of the empathy experience (Morelli et al., 2015).

#### Empathy and efficacy

In prior work, researchers have demonstrated that some individuals are more empathically accurate than others, and this accuracy has real-life consequences for relationships (Ickes, 1993; Sened et al., 2017). Not only does actual empathic accuracy matter, but also experimental research has demonstrated that perceptions of empathic accuracy can affect people’s willingness to empathize with others, regardless of actual empathic accuracy (Cameron et al., 2019). People who believe that their empathizing is more accurate and effective are less likely to avoid empathy opportunities. Thus, in the current study, we asked participants how subjectively difficult their empathy experiences were and how (subjectively) confident they were about the accuracy of their empathy. The extent to which these perceptions of accuracy map onto actual accuracy is unknown.

However, participants’ sense of confidence and difficulty when empathizing were important factors for their empathy and broader experience. Confidence and difficulty during empathy experiences impacted subjective well-being (see Table 1). Further, participants reported empathizing to a greater extent as their confidence increased, *b* = 0.54, *SE* = 0.04, *t*(998) = 14.76, *p* < .001, *r* = .42 (a medium-size effect), and to a lesser extent as difficulty increased, *b* = −0.21, *SE* = 0.03, *t*(982) = −8.20, *p* < .001, *r* = .25 (a small effect).

**Table 1.** Within- and Between-Subjects Effects From Models Predicting Well-Being From Different Aspects of Daily Empathy

Predictor	Within-subjects effects				Between-subjects effects			
	Test statistic	Adjusted *p*	*b* (*SE*)	Effect size	Test statistic	Adjusted *p*	*b* (*SE*)	Effect size
Empathy opportunity	*z* = 6.72	< .001	0.18 (0.03)	.08	*z* = 4.39	< .001	1.81 (0.41)	.27
Empathy (yes/no)	*z* = 3.08	.003	0.25 (0.09)	.09	*z* = 0.96	.340	0.34 (0.36)	.06
Emotion sharing (yes/no)	*z* = 2.5	.015	0.21 (0.09)	.08	*z* = 1.29	.211	0.43 (0.33)	.09
Perspective taking (yes/no)	*z* = −1.24	.214	−0.10 (0.08)	.04	*z* = 1.29	.211	0.43 (0.33)	.09
Compassion (yes/no)	*z* = 2.02	.050	0.26 (0.13)	.06	*z* = 3.29	.002	1.31 (0.40)	.22
Extent of emotion sharing	*t*(875) = 3.25	.002	0.08 (0.03)	.11	*t*(875) = 3.86	< .001	0.35 (0.09)	.26
Extent of perspective taking	*t*(804) = 2.96	.004	0.06 (0.02)	.10	*t*(804) = 4.46	< .001	0.35 (0.08)	.30
Extent of compassion	*t*(938) = 5.61	< .001	0.16 (0.03)	.18	*t*(938) = 4.62	< .001	0.50 (0.11)	.31
Extent of personal distress	*t*(1152) = −9.71	< .001	−0.14 (0.01)	.28	*t*(1152) = −6.83	< .001	−0.38 (0.06)	.42
Extent of empathy^a^	*t*(991) = 5.73	< .001	0.17 (0.03)	.18	*t*(991) = 5.63	< .001	0.55 (0.10)	.36
Empathy difficulty	*t*(965) = −4.61	< .001	−0.12 (0.03)	.15	*t*(965) = −3.19	.002	−0.25 (0.08)	.21
Empathy confidence	*t*(969) = 6.98	< .001	0.26 (0.04)	.22	*t*(969) = 4.48	< .001	0.49 (0.11)	.29
Extent of empathy received	*t*(614) = 5.16	< .001	0.20 (0.04)	.20	*t*(614) = 5.37	< .001	0.56 (0.10)	.37
Valence of observed emotion	*t*(1132) = 10.31	< .001	0.15 (0.01)	.29	*t*(1132) = 11.69	< .001	0.17 (0.01)	.31
Opportunity to receive empathy	*z* = 1.28	.213	0.04 (0.03)	.02	*z* = 2.34	.025	1.12 (0.48)	.15

Note: The results shown here are derived from multilevel models including a random intercept for both participant and survey day. Each predictor was run in a separate model, and *p* values were subsequently adjusted together to control the false-discovery rate. Effect sizes are given as *r* values. ^a^This variable includes emotion sharing, perspective taking, and compassion.

### Correlates of empathy

#### Subjective well-being

Empathy was generally associated with higher subjective well-being, but the relationship was complex (see Table 1). Overall, participants reported fairly high levels of subjective well-being (on a scale from 1 to 7; *M* = 5.02, *SD* = 1.59, 95% CI = [4.99, 5.06]). They reported higher subjective well-being when they had experienced an empathy opportunity (*M* = 5.48, *SD* = 1.47, 95% CI = [5.41, 5.56]) compared with when they had not (*M* = 4.92, *SD* = 1.60, 95% CI = [4.88, 4.96]), *b* = 0.18, *SE* = 0.03, *t*(6615) = 6.72, *p* < .001, *r* = .08. Similarly, actually experiencing empathy, *b* = 0.25, *SE* = 0.08, *t*(1231) = 3.08, *p* = .002, *r* = .09, and the extent of empathy experienced, *b* = 0.17, *SE* = 0.03, *t*(992) = 5.73, *p* < .001, *r* = .18, were further predictive of increased well-being.

**Fig. 4.**
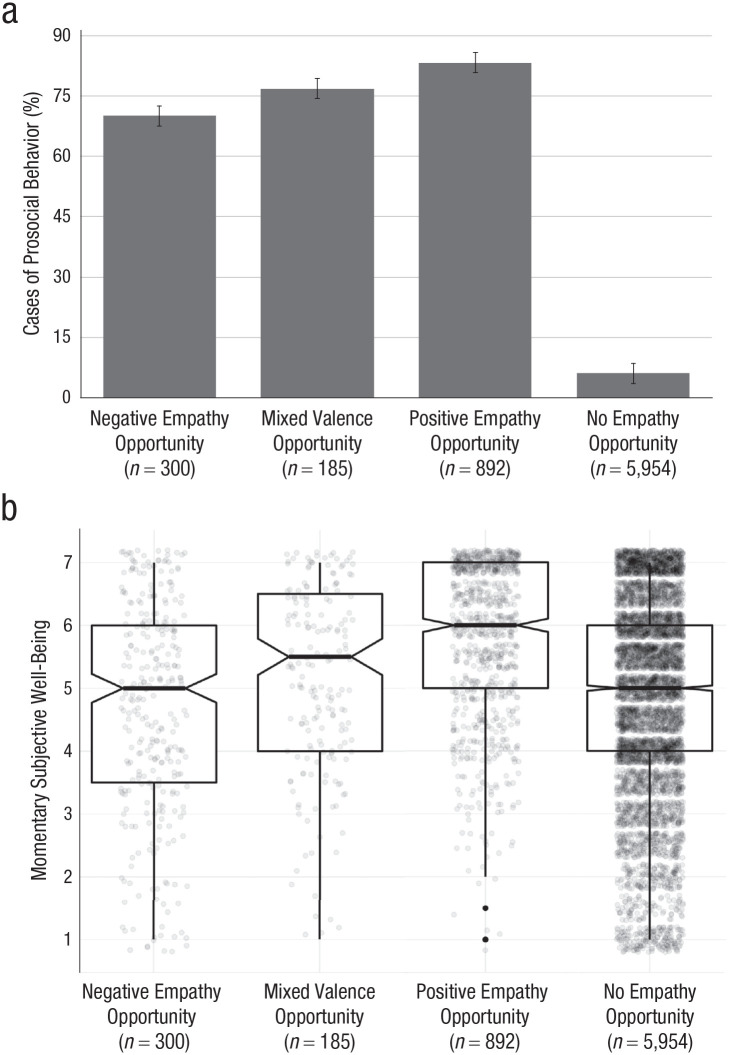
Mean rate of reported prosocial behavior (a) and subjective well-being (b) in the 15-min windows in which participants had negative, mixed, positive, or no opportunities to empathize. In (a), error bars indicate 95% confidence intervals for the mean estimates. In (b), boxes show where 50% of the data lies, the horizontal lines indicates means, and notches show 95% confidence intervals for the means. Whiskers indicate outlier boundaries. Dots represent individual observations.

However, the positive impact of empathy opportunities was associated with the valence of the target emotion. Unlike empathy opportunities for positive emotions (*M* = 5.85, *SD* = 1.24, 95% CI = [5.78, 5.93]) and mixed emotions (*M* = 5.10, *SD* = 1.48, 95% CI = [4.89, 5.32]), empathy opportunities for negative emotions (*M* = 4.63, *SD* = 1.69, 95% CI = [4.44, 4.82]) were associated with lower levels of well-being than average (see Fig. 4).

Further, not all aspects of everyday empathy were associated with increased subjective well-being. In particular, the extent to which participants found empathy difficult was negatively correlated with subjective well-being at both the within-subjects level, *b* = −0.12, *SE* = 0.03, *t*(965) = −4.59, *p* < .001, *r* = .15, and between-subjects level, *b* = −0.24, *SE* = 0.08, *t*(212) = −3.07, *p* = .002, *r* = .21. Extent of personal distress was similarly associated with decreased well-being both within subjects, *b* = −0.14, *SE* = 0.01, *t*(1152) = −9.71, *p* < .001, *r* = .28, and between subjects, *b* = −0.39, *SE* = 0.06, *t*(222) = −6.89, *p* < .001, *r* = .42, the latter being a medium-size effect. These data suggest that, although many components of empathy are associated with increased subjective well-being in daily life, others are associated with decreased well-being, and the overall effect is importantly influenced by the valence of the target emotion.

#### Prosocial behavior

Considerable research has found that empathy for negative emotions promotes helping behavior (Davis et al., 2015; Eisenberg & Miller, 1987). More recently, researchers have reported that empathy for positive emotions may do the same (Andreychik & Lewis, 2017; Morelli et al., 2014; Telle & Pfister, 2016). We observed strong correlational support for both of these effects (see Fig. 4), finding that several aspects of everyday empathy were correlated with prosocial behavior in daily life (see Table 2). Reported empathy opportunities showed a large correlation with reported prosocial behavior in the same 15-min window, and prosocial behaviors were much more likely when participants reported an empathy opportunity compared with no empathy opportunity, *b* = 4.41, *SE* = 0.15, *z* = 33.38, *p* < .001, *r* = .77.^2^ Experiencing empathy in the presence of an opportunity was further predictive of prosocial behavior, *b* = 1.30, *SE* = 0.22, *z* = 5.85, *p* < .001, *r* = .34 (a medium-size effect). Interestingly, opportunities to receive empathy were also predictive of prosocial behavior, *b* = 2.11, *SE* = 0.10, *z* = 20.70, *p* < .001, *r* = .50 (a large effect and a relatively unexplored link in the literature that merits further investigation).

**Table 2.** Within- and Between-Subjects Effects From Models Predicting Prosocial Behavior From Different Aspects of Daily Empathy

Predictor	Within-subjects effects				Between-subjects effects			
	Test statistic	Adjusted *p*	*b* (*SE*)	Effect size	Test statistic	Adjusted *p*	*b* (*SE*)	Effect size
Empathy opportunity	*z* = 33.38	< .001	4.41 (0.13)	.77	*z* = 24.16	< .001	5.73 (0.24)	.85
Empathy (yes/no)	*z* = 5.85	< .001	1.30 (0.22)	.34	*z* = 0.19	.853	0.07 (0.38)	.02
Emotion sharing (yes/no)	*z* = 2.98	.007	0.73 (0.25)	.20	*z* = 2.30	.035	0.83 (0.36)	.22
Perspective taking (yes/no)	*z* = 2.19	.047	0.51 (0.23)	.14	*z* = 2.30	.035	0.83 (0.36)	.22
Compassion (yes/no)	*z* = 2.56	.021	0.90 (0.35)	.24	*z* = 2.56	.035	1.12 (0.44)	.30
Extent of emotion sharing	*t*(875) = 1.79	.100	0.16 (0.09)	.05	*t*(875) = 2.79	.013	0.27 (0.10)	.07
Extent of perspective taking	*t*(804) = −0.03	.976	−0.003 (0.08)	.10	*t*(804) = 3.81	< .001	0.34 (0.09)	.09
Extent of compassion	*t*(938) = 3.50	.002	0.37 (0.11)	.10	*t*(938) = 1.91	.070	0.23 (0.12)	.06
Extent of personal distress	*t*(1152) = −1.04	.346	−0.05 (0.05)	−.01	*t*(1152) = −1.59	.121	−0.10 (0.06)	−.03
Extent of empathy^1^	*t*(991) = 2.19	.047	0.23 (0.11)	.06	*t*(991) = 3.71	< .001	0.41 (0.11)	.11
Empathy difficulty	*t*(965) = −0.38	.76	−0.04 (0.10)	−.01	*t*(965) = 2.11	.048	−0.18 (0.08)	−.05
Empathy confidence	*t*(969) = 1.97	.074	0.27 (0.14)	.08	*t*(969) = 2.28	.035	0.28 (0.12)	.08
Extent of empathy received	*t*(614) = 3.04	.006	0.38 (0.13)	.11	*t*(614) = 1.84	.076	0.21 (0.12)	.06
Valence of observed emotion	*t*(1132) = 1.53	.159	0.08 (0.05)	.02	*t*(1132) = 2.96	.009	0.13 (0.04)	.04
Opportunity to receive empathy	*z* = 20.70	< .001	2.11 (0.10)	.50	*z* = 15.13	< .001	5.69 (0.38)	.84

Note: The results shown here are derived from multilevel models including a random intercept for both participant and survey day. Each predictor was run in a separate model, and *p* values were subsequently adjusted together to control the false-discovery rate. Effect sizes are given as *r* values. ^a^This variable includes emotion sharing, perspective taking, and compassion.

Prosocial behavior was often elicited in the presence of positive and mixed emotions (see Fig. 4). In fact, positive empathy opportunities were a little more predictive of prosocial behavior than negative opportunities, *b* = 0.54, *SE* = 0.19, *z* = 2.79, *p* = .005, *r* = .15. Further—although participant-centered valence did not predict prosocial behavior, *b* = 0.08, *SE* = 0.05, *z* = 1.53, *p* = .159, *r* = .02—participants who typically experienced more positively valenced empathy opportunities were slightly more likely to report prosocial behaviors in a given survey, *b* = 0.13, *SE* = 0.04, *z* = 2.96, *p* = .009, *r* = .04 (a between-subjects effect). In sum, this set of results underscores the fact that perceived empathy opportunities are associated with prosocial behavior not only in the face of suffering but also when positive and mixed emotions are observed.

#### Trait versus state empathy predicting well-being and prosocial acts

Having described the association between daily empathy and well-being and between daily empathy and prosocial behavior, we conducted preregistered analyses to investigate whether trait empathy predicted these outcomes. After we controlled the FDR (Benjamini & Hochberg, 2007), there were no significant associations between trait empathy and daily prosocial behavior (all *p*s > .05; see Table S1 at https://osf.io/zd6wv/). Trait empathy also did not predict increased subjective well-being in daily life (all *p*s > .05; see Table S2 at https://osf.io/zd6wv/). If anything, we found evidence for the opposite: The associations between lower subjective well-being in daily life and both higher personal distress (measured via a subscale of the Interpersonal Reactivity Index), *b* = −0.47, *SE* = 0.10, *t*(236) = −4.46, *p* < .001, *r* = .28, and higher empathy (measured via the Empathy Index), *b* = −0.33, *SE* = 0.11, *t*(236) = −2.89, *p* = .018, *r* = .19, were in fact associated with *lower* subjective well-being in daily life—though effects were small. Overall, higher trait empathy did not seem to be associated with more prosocial behaviors or higher well-being in momentary assessments.

However, many aspects of empathy in everyday life were positively associated with momentary assessments of prosocial behavior, and these associations held after adjustments for multiple comparisons (see Table 1). In addition, many aspects of daily empathy were associated with increased subjective well-being at both the within- and between-subjects levels (see Table 2). These effects highlight the importance of empathy and support prior reports that experience sampling may uncover associations with well-being not captured by more global trait measures (e.g., Runyan et al., 2019).

#### Empathic facilitation

Moral licensing is the phenomenon whereby behaving morally can lead to future immoral behaviors (Merritt et al., 2010). Although the robustness of the effect has been questioned (Blanken et al., 2015; Kuper & Bott, 2019), there is evidence for its existence in daily life (Hofmann et al., 2014). Whether there is a similar effect with empathy—or whether empathy tends to promote more empathy (i.e., empathy facilitation)—is unknown. To explore this question, we examined whether aspects of empathy at Time 1 would predict the likelihood of empathy opportunities or empathy itself at Time 2. These surveys occurred 90 min apart on average.

For the most part, after FDR correction, there was little evidence of any licensing or facilitation effects. However, noticing opportunities to receive empathy at Time 1 predicted noticing opportunities to provide empathy, *b* = 0.28, *SE* = 0.10, *z* = 2.71, *p* = .043, *r* = .08, and act prosocially, *b* = 0.23, *SE* = 0.11, *z* = 2.09, *p* = .037, *r* = .06, at Time 2, although both effects were very small. That is, when participants had an emotional experience and felt that someone had an opportunity to empathize with them, they were more likely to report a prosocial behavior and more likely to report an opportunity to empathize with someone else on the next survey they answered.

Although perceiving an opportunity to empathize at Time 1 did not predict noticing another opportunity at Time 2, *b* = −0.02, *SE* = 0.09, *z* = −0.23, *p* = .817, *r* = −.01, missing an opportunity at Time 1 did predict noticing an opportunity at Time 2, *b* = 0.32, *SE* = 0.12, *z* = 2.64, *p* = .008, *r* = .09. In other words, when participants reported no empathy opportunity but reported observing the emotions of another person, they were more likely to report an empathy opportunity at their next survey. No aspects of empathy at Time 1 predicted whether participants actually engaged in empathy given the opportunity at Time 2 (all *p*s > .05). In sum, we did not find robust support for empathic licensing, instead finding preliminary evidence that receiving empathy or missing an opportunity to empathize may facilitate future empathy opportunities.

### Who feels empathy: demographics of daily empathy

In addition to conducting the analyses above, we tested previously reported demographic differences in empathy. In our only preregistered demographic test, sex was a small but significant predictor of empathy. Women scored higher than men on trait empathic concern, *b* = 0.36, *SE* = 0.09, *t*(232) = 4.17, *p* < .001, *r* = .26. Congruent with this, results showed that women reported feeling compassion, *b* = 0.30, *SE* = 0.11, *t*(187) = 2.75, *p* = .007, *r* = .20, and sharing emotions, *b* = 0.30, *SE* = 0.13, *t*(166) = 2.26, *p* = .025, *r* = .17, to a greater extent than men in everyday life. Women also reported higher confidence that their empathy was accurate, *b* = 0.29, *SE* = 0.10, *t*(180) = 2.80, *p* = .006, *r* = .20, which may in part explain their increased willingness to empathize (Cameron et al., 2019).

We next performed nonpreregistered tests on three other previously reported demographic differences. We examined whether compassion varied across income levels (Stellar et al., 2012) and found mixed results. Higher income was not associated with decreased trait empathic concern, *b* = −0.04, *SE* = 0.05, *t*(232) = −0.73, *p* = .466, *r* = .05, or decreased extent of compassion in daily life, *b* = 0.06, *SE* = 0.06, *t*(200) = 0.88, *p* = .380, *r* = .06. However, higher income was associated with a lower proportion of feeling compassion in the presence of an empathy opportunity, *b* = −0.04, *SE* = 0.02, *t*(208) = −2.34, *p* = .020, *r* = .16, indicating that although higher income participants felt compassion to a similar extent, they felt it less often.^3^

We replicated previous reports that religiosity is correlated with increased empathy (Watson et al., 1984). Higher religiosity weakly predicted increased likelihood of reporting an empathy opportunity, *b* = 0.25, *SE* = 0.07, *z* = 3.47, *p* = .006, *r* = .07, and predicted increased extent of perspective taking, *b* = 0.19, *SE* = 0.06, *t*(166) = 3.29, *p* = .007, *r* = .25, in everyday life. After correction, religiosity was not associated with any other daily empathy or trait empathy measures (all *p*s > .05), although it is possible that the true effects were smaller than our study was powered to detect (*d* = 0.33, *r* = .16).

Prior work has found that liberals experience more empathy (Hasson et al., 2018) than conservatives for a wider range of targets (Waytz et al., 2019) in certain circumstances. However, we found that after adjustment, political orientation was related to only the fantasy subscale of the Interpersonal Reactivity Index, *b* = −0.09, *SE* = 0.03, *t*(236) = −3.19, *p* = .018, *r* = .20, in that more conservative participants reported empathizing less with fiction. We did not find any other associations between empathy and political orientation at the trait or state level (all *p*s > .05). We note, however, that prior effects may be context specific (Lucas & Kteily, 2018). Further, our study was powered to detect effects (*d*s) only as small as 0.33 (*r* = .16) at the trait level, so it is possible that there are true effects that were too small for us to detect.

## Discussion

The majority of research on empathy has focused on negative emotions—typically of strangers and typically in laboratory settings. However, in everyday life, empathy was more often reported in response to positive emotions, not negative emotions, and participants empathized to a greater extent as emotions became more positive. Although these results may be influenced by reporting biases, they are consistent with the relative frequency of emotions experienced in daily life; positive emotions, such as excitement and enthusiasm, are experienced approximately 3 times more frequently than negative emotions, such as disgust, anger, and fear (Zelenski & Larsen, 2000).

Empathy was most often reported in response to close others. This effect may be due to mere availability because many people likely have more social interactions within existing relationships than with strangers. However, empathy also may be biased in favor of close others (Cikara et al., 2011). Supporting this possibility, our results showed that individuals empathized to a greater extent as closeness increased.

These findings have implications for our understanding of empathy as a motivated phenomenon (Zaki, 2014). The emerging work on lack of motivation to empathize might be related, in some part, to most lab studies of empathy involving the negative emotions of strangers. Whereas some work suggests that people might avoid both positive and negative empathy (Cameron et al., 2019), empathy for strangers might be especially unmotivating (Ferguson et al., 2020). In future lab studies on motivation to empathize, researchers should consider including a range of expressed emotions and including empathy targets who are close to the participant, not just strangers.

In prior work, researchers have suggested that dissociable components of empathy interact in real-life interactions (Morelli et al., 2014; Zaki & Ochsner, 2012). The current study shows that emotion sharing, compassion, and perspective taking are reported together almost all of the time and are rarely reported in isolation. Although these components of empathy can be theoretically differentiated, our data suggest that they are typically experienced together by most people in most daily situations.

Finally, we examined various established demographic findings about empathy to see which findings hold across people’s experiences in daily life. Some established effects were replicated—for example, both women and religious participants tended to report experiencing empathy more often than men and the nonreligious. However, other relationships did not replicate in the context of daily life; we found a weak relationship between compassion and income and little to no relationship between empathy and political orientation—although the true effects of income and politics on empathy may be smaller than we were able to detect given our statistical power.

### Limitations

#### Ground truth

Experience sampling allows us to get closer to the temporal, emotional, and social context of empathy, but it is still self-report data. The number of empathy opportunities and true ratios of positive and negative opportunities may vary from those reported—indeed, participants appeared selective in which observed emotions were perceived as empathy opportunities, consistent with motivational accounts of empathy (Cameron et al., 2019; de Vignemont & Singer, 2006; Zaki, 2014). However, truly objective measures of empathy opportunities would be difficult to obtain, given that empathy cues in the environment may not be attended to. Furthermore, feeling empathy may be best captured via self-report, given that it is an internal and subjective phenomenon.

#### Representativeness

Our sample was quota matched to census data on six key demographics, making our results more representative than is typical. Because the sample is not random, representativeness cannot be assumed on other demographics. For example, to join the study, participants were required to have a smartphone. However, 81% of U.S. adults had a smartphone as of 2019 (Pew Research Center, 2021), and the results from our lowest income participants mirrored those from the entire sample. The generalizability of our findings to other populations, especially non–Western, educated, industrialized, rich, and democratic (WEIRD) populations, remains to be demonstrated.

#### Training and fatigue

Potentially, repeatedly responding to surveys on empathy might have trained participants to notice empathy opportunities. However, there was only one significant difference on trait empathy questionnaires at baseline compared with the final experience-sampling survey (all *p*s > .05), and empathy itself did not become more prevalent over the course of the week. Empathy opportunities were reported slightly less frequently as the week progressed, suggesting that individuals may have become decreasingly inclined to report empathy. However, because we nested results within survey day, this shift should minimally affect our results.

#### Definitions and demand

We chose to supply a definition of empathy to our participants to reduce noise from varying lay theories of empathy (Hall et al., 2021). By defining empathy as a process involving three related but distinct experiences, however, we may have introduced demand, resulting in participants inflating reports of the co-occurrence of all three processes or otherwise influencing participants’ reports (although similar results were reported for participants’ own empathy and empathy received). Future work should replicate these findings with different definitions of empathy.

### Conclusion

The current study has shaped how we think about empathy. People readily empathize when they recognize the opportunity but often notice other people’s emotions without flagging them as empathy opportunities. In daily life, empathy is often elicited by positive rather than negative emotions and by emotional expressions of close others rather than strangers. These caveats are critical to consider in future experiments because they alter the experience of empathy itself and likely moderate the effects of empathy on subjective well-being and prosocial behavior. If our experiments are to inform us about the causal role of empathy outside of the lab, they must in turn be informed by how empathy is actually experienced in everyday life.

## Transparency

*Action Editor:* Mark Brandt

*Editor:* Patricia J. Bauer

*Author Contributions*

G. J. Depow and M. Inzlicht conceived the idea for the project. G. J. Depow collected the data in conjunction with a survey company and an experience-sampling company. Z. Francis and M. Inzlicht provided critical support. G. J. Depow analyzed the data; Z. Francis provided guidance and created the figures. G. J. Depow wrote the first draft of the manuscript with advice from M. Inzlicht. Z. Francis and M. Inzlicht provided critical revisions. All the authors revised the manuscript in response to reviews and approved the final manuscript for submission.

**Open Practices:** All data, code, and materials have been made publicly available via OSF and can be accessed at https://osf.io/y3ud7. The design and analysis plan were preregistered at https://osf.io/aeqgn. Deviations from the preregistration (preregistered analyses not reported here as well as additional nonpreregistered analyses conducted) are noted in the text. This article has received the badges for Open Data, Open Materials, and Preregistration. More information about the Open Practices badges can be found at http://www.psychologicalscience.org/publications/badges.

## Notes

1. The richness of the data set and diversity of preregistered questions mean that some of the preregistered questions are best addressed in separate articles. Importantly, other articles using these data will be nonoverlapping, addressing distinct preregistered questions (see Table S3 in at https://osf.io/zd6wv/).

2. This estimate implies that prosocial acts are 85 times more likely when an empathy opportunity is reported. Empathy opportunities were defined to participants as “any situation where you see/hear about an event that probably caused an emotional response for the person involved,” and it is not clear whether the opportunity precedes or follows the prosociality. However, this effect size is implausibly large and may be inflated by overfitting and demand.

3. Income was also unassociated with the binomial and extent of perspective taking and emotion sharing in daily life. Further, it was unassociated with reports of prosocial behavior in daily life.
